# Pharmacokinetics and tolerability of single-dose enteral cannabidiol and cannabidiolic acid rich hemp in horses (*Equus caballus*)

**DOI:** 10.3389/fvets.2024.1356463

**Published:** 2024-04-12

**Authors:** Alexander C. S. Thomson, Taralyn M. McCarrel, Alexander Zakharov, Beatriz Gomez, Alex Lyubimov, Wayne S. Schwark, Martha F. Mallicote, Diego A. Portela, Amber L. Bisiau, Joseph J. Wakshlag

**Affiliations:** ^1^Department of Comparative, Population, and Diagnostic Medicine, College of Veterinary Medicine, University of Florida, Gainesville, FL, United States; ^2^Department of Large Animal Clinical Sciences, College of Veterinary Medicine, University of Florida, Gainesville, FL, United States; ^3^Department of Pharmacology, College of Medicine, University of Illinois, Chicago, IL, United States; ^4^Department of Molecular Medicine, College of Veterinary Medicine, Cornell University, Ithaca, NY, United States; ^5^Department of Clinical Sciences, College of Veterinary Medicine, Cornell University, Ithaca, NY, United States

**Keywords:** cannabidiol, pharmacokinetic, horse, cannabidiolic acid, activity, gastrointestinal

## Abstract

The pharmacokinetics and tolerability of cannabinoids and their metabolites were determined in eight horses after enteral administration of a commercial CBD/CBDA-rich hemp oil product. Each horse was administered 2 mg/kg or 8 mg/kg CBD/CBDA or no treatment in a randomized cross-over design. Serial serum samples collected over 48 h were analyzed by high performance liquid chromatography with tandem mass spectrometry. Plasma chemistry analysis was performed at 0 h and 24 h. Vital parameters, pedometry, and blinded mentation and gait evaluations were recorded at intervals up to 24 h. Manure production and gastrointestinal transit time were tracked for 48 h after oil administration. The median maximal concentration of CBD and CBDA were 5.2 and 36.95 ng/mL in the 2 mg/kg group, respectively; and 40.35 and 353.56 ng/mL in the 8 mg/kg group. The median half-life of elimination was not calculated for the 2 mg/kg CBD treatment due to lack of time points above the lower quantifiable limit beyond the Cmax while it was 7.75 h in the 8 mg/kg group. CBDA absorption was biphasic. Pharmacokinetic parameters for tetrahydrocannabinol, tetrahydrocannabinolic acid, cannabigerolic acid, and 7-carboxy cannabidiol are also reported. No significant differences in any of the measured tolerability parameters were demonstrated between treatment groups. Single-dose enteral administration of CBD/CBDA-rich hemp extract up to 8 mg/kg does not appear to produce neurologic, behavioral, or gastrointestinal effects in horses.

## 1 Introduction

The endocannabinoid system is a complex yet highly conserved cell signaling pathway found in all chordates, consisting of endocannabinoids, cannabinoid receptors, and metabolizing enzymes (1). Endocannabinoids are eicosanoids synthesized from the polyunsaturated fatty acids present in the lipid membranes of all cells. The most prevalent of the six identified endocannabinoids are anandamide and 2-arachidonoylglycerol, which serve as ligands for the G-protein coupled receptors CB_1_ and CB_2_ (2). These receptors are found throughout the body, with CB1 highly concentrated in the central nervous system and CB2 found more in other organ systems and the immune system, where they play diverse roles from regulating appetite, neuronal action potentials to mediating immune responses. Endocannabinoids are metabolized rapidly in the synapse by the enzymes fatty acid amide hydrolase and monoacylglycerol lipase (3).

Phytocannabinoids are chemicals found in *Cannabis sativa* that share structural similarity to the endocannabinoids and may interact with cannabinoid receptors or enzymes in the endocannabinoid metabolic pathway. At least 113 different phytocannabinoids have been identified in *Cannabis*; the most abundant across strains are tetrahydrocannabinolic acid (THCA), cannabidiolic acid (CBDA), cannabigerolic acid (CBGA), cannabidivarinic acid (CBDVA), and cannabichromenic acid (CBCA), which during extraction are often decarboxylated to the neutral cannabinoids cannabidiol (CBD), Δ^9^-tetrahyrocannbinol (THC), cannabigerol (CBG), cannabidivarin (CBDV), and cannabichromene (CBC) (4). Phytocannabinoids act as agonists, inverse agonists or antagonists with cannabinoid receptors, metabolic enzymes, and other receptors/channels such as those in the transient receptor potential cation channel family (5, 6). Some phytocannabinoids, including CBD, show promise in therapeutic applications in veterinary species (7–11).

CBD is one of the most abundant phytocannabinoids extracted from *Cannabis sativa*, accounting for as much as 40% of the plant’s extract (12). While CBD has low affinity for CB_1_ and CB_2_ cannabinoid receptors (13), it functions as an endocannabinoid modulator by inhibiting fatty acid amide hydrolase and anandamide reuptake, as well as other arachidonic acid metabolizing enzymes (14). A variety of additional mechanisms of action make CBD an interesting candidate as an analgesic agent. CBD activates and desensitizes transient receptor potential vanilloid 1 (TRPV1) channels (5), found in abundance in central nervous system pain pathways. CBD also inhibits glutamate release and suppresses the production of tumor necrosis factor-alpha (15, 16). CBD may work synergistically with other analgesic agents, such as opioids, inhibiting their metabolism via the cytochrome p450 system (17). CBDA can work at similar receptor systems as CBD and is a selective inhibitor of cyclooxygenase-2, a key enzyme involved in inflammation (18). In recent years, interest in cannabinoid medicine has increased rapidly, among both the medical and veterinary communities and the general public, spurred by the drawbacks of conventional analgesic medications such as opioids and non-steroidal anti-inflammatory drugs (19, 20). Cannabinoids have been promoted in both human and veterinary medicine as alternatives or adjuncts to conventional medications in the treatment of pain, seizures, and a variety of other disorders (9, 10). Oral CBD formulations are already marketed to and used by horse owners, often without veterinary oversight. However, only a few published studies exist on the pharmacokinetics, pharmacodynamics, or safety of cannabinoids in horses (21–26). Due to the scarcity of published data in horses, clinical recommendations have often relied on anecdotal experience and extrapolation of data from other species. Oral nutraceuticals show significant inter-species differences in oral bioavailability, potentially leading to significant over- or underdosing when extrapolating doses from one species to another (27). CBD isolates and CBD rich hemp products available for horses come in a wide range of concentrations, formulations, and purity, but few evidence-based guidelines exist for their administration. In addition, CBD, THC, and their metabolites are subject to regulatory control under certain racing jurisdictions and competition horse associations. Further research may demonstrate CBD’s utility as an adjunctive analgesic agent. Unfortunately, no such research can proceed meaningfully without a basic knowledge of the pharmacokinetic, tolerability and pharmacodynamics of cannabinoids in full spectrum hemp extracted products which may be different from isolates of cannabinoids (28).

The primary aim of this study was to determine the basic enteral non-compartmental pharmacokinetics of a single dose of CBD/CBDA-rich full spectrum hemp oil in fit, exercised horses similar to what has been done in dogs at 2 and 8 mg/kg (9). The secondary aims of this study were to evaluate if the tested doses of CBD/CBDA produced adverse effects on clinical neurologic status, gastrointestinal transit time, clinicopathologic variables, or altered spontaneous ambulation in horses confined to stalls.

## 2 Materials and methods

### 2.1 CBD/CBDA full spectrum oil

CBD/CBDA-rich hemp oil was administered by nasogastric tube in a proprietary oil formulation (Hemp CBD + CBDA Oil, ElleVet Sciences, ME, USA). Compositional analysis of the oil was performed by liquid chromatography by a certified ISO/IEC 17025 third-party laboratory (ProVerde Laboratories, MA, USA). The oil contained 27.73 mg/mL CBD, 34.10 mg/mL CBDA, 1.32 mg/mL Δ^9^-THC, 1.27 mg/mL THCA, 0.35 mg/mL CBG, 0.89 mg/mL CBGA, 1.1 mg/mL CBC, and trace levels of CBDV and CBN. The oil contained no tetrahydrocannabivarin (THCV), Δ^8^-tetrahydrocannabinol (Δ^8^-THC), or exo-tetrahydrocannabinol (exo-THC). The oil product used passed all mycotoxin, heavy metal, microbial, pesticide and solvent contamination tests and complies with USDA certified hemp GMP protocols for hemp production.

### 2.2 Animals

Eight healthy Thoroughbred horses from a dedicated research herd, two castrated males and six intact females, 3–10 years of age, weighing 526.5 ± 33.4 kg and of ideal body condition were included in the study. Horses were trained for at least 2 months on a high-speed treadmill (Mustang 2200, Graber AG, Switzerland) to achieve fitness representative of Thoroughbred racehorses prior to commencing the study. The standard training regimen was 0.6 km at 4 m/s then 2 km at 8 m/s and 0.6 km at 4 m/s 3 days per week. Prior to each trial horses had to demonstrate adequate fitness by running 1.6 km at 13.5 m/s with warm-up and cool-down of 0.96 km at 4 m/s. Following the fitness test, heart rate was monitored every 5 min and must be below 50 beats per minute within 40 min of concluding the test for the horse to prove fitness. The standard training regimen was maintained during the washout periods but horses were not exercised during the 48-h data collection window for each trial. No concurrent medications or supplements were permitted during the data collection or intervening washout periods. Horses were housed in matted, 13.4 m^2^ stalls on data collection days and returned to their normal outdoor pastures during the intervening washout periods. During the study period, horses were allowed free access to water and coastal Bermuda hay and fed a proprietary pelleted feed once per day in the morning, approximately 2 h prior to treatment. All experimental protocols were reviewed and approved by the University of Florida Institutional Animal Care and Use Committee (protocol 201808925).

### 2.3 Animal treatments and blood sampling

The study was conducted in three 48-h trials in a randomized cross-over design, each separated by a minimum 2-week washout period during which time the horses were returned to their normal pastures. Each horse completed a no treatment control (water only) trial as well as a 2 mg/kg and 8 mg/kg CBD/CBDA-rich oil dose trial. The dose was calculated based on body weight and a total combined CBD/CBDA concentration in the oil formulation of 62.0 mg/mL (28 mg/mL CBD + 34 mg/mL CBDA). Approximately 2 h after feeding on the first morning of each trial, barium spheres were delivered in approximately 2 L of water via nasogastric tube. Then, in the treatment groups, CBD/CBDA rich hemp oil (Ellevet Sciences, Portland, ME) was delivered via nasogastric tube to ensure accuracy in dosing. The nasogastric tube was flushed again with approximately 2 L of water to flush in residual oil. Nasogastric tubes, water buckets, and funnels were designated as CBD/CBDA treatment or no treatment throughout the study to ensure there was no risk of contamination of control treated horses with CBD/CBDA oil.

Blood was collected by jugular venipuncture into 10 mL red top tubes immediately prior to CBD/CBDA oil administration and at 0.5, 1, 1.5, 2, 3, 4, 8, 12, 24, and 48 h after administration. Blood samples were allowed to coagulate for 1 h and then centrifuged, and the serum was separated into new tubes and stored at −80°C until analysis.

### 2.4 Cannabinoids analysis in horse serum by LC–MS/MS

Analysis was performed using an exploratory (fit-for-purpose) method for measurement of thirteen cannabinoids and their metabolites at the Toxicology Research Laboratory, University of Illinois at Chicago. The reference standards for CBD and CBDA were obtained from Restek Corporation (Bellefonte, PA); all other reference and internal standards were obtained from Cerilliant Corporation (Round Rock, TX). Cannabinoids (CBD, CBDA, THC, THCA, CBN, CBC, CBG, and CBGA) and their metabolites (11-OH-THC, 7-OH-CBD, 7-COOH-CBD, COOH-THC, and COOH-THC-Glu) concentration in horse serum was determined using high performance liquid chromatography with tandem mass spectrometry (LC–MS/MS) (Nexera X2 and MS 8050, Shimadzu Corp., Kyoto, Japan).

Horse serum (40 μL) was mixed with 20 μL of internal standards (100 ng/mL of CBD-d3, THC-d3, THCA-d3, 7-COOH-CBD-d3, 7-OH-CBD-d5, 11-OH-THC-d3, COOH-THC-d9, and COOH-THC-Glu-d3 in 50% methanol) in a 96 well plate. Proteins were precipitated and compounds were extracted by adding 100 μL of ice-cold acetonitrile to the samples, then vortexing for 1–2 min and centrifuging at 4,000 rpm (2,300 *g*) for 10 min at 4°C. Supernatants (80 μL) were mixed with 80 μL of water in a different 96 well plate, and centrifuged again. The processed samples (10 μL) were injected into Waters Atlantis T3 HPLC column (3 μm 2.1 × 50 mm) with a guard cartridge (Waters VanGuard Atlantis T3) coupled to LC–MS/MS. The column was equilibrated with mobile phase A (0.1% formic acid in water) and mobile phase B (acetonitrile) at 50% B. The compounds were eluted by a linear gradient from 50% B to 95% B over 6 min, and then held at 95% B for 1 min. Subsequently, the column was re-equilibrated at initial composition for 1 min. Flow rate was 0.3 mL/min. The autosampler and column temperature were set at 4 and 30°C, respectively. The compounds were detected in electrospray ionization positive and/or negative mode as described in the Supplementary Table 1. Interface voltage was 4 kV and −3 kV, respectively. Interface, desolvation line, and heat block temperature were 300, 200, and 400°C, respectively. Nebulizing, heating, and drying gas flow were 2.7, 5, and 5 L/min, respectively.

Concentrations of cannabinoids were calculated by LabSolutions software (Shimadzu Corp., Kyoto, Japan) using a quadratic calibration curve with 1/c^2^ weighing based on relative response (peak area of cannabinoids/peak area of internal standards). The calibration curve range, lower limit of detection and quantitation in horse serum is shown in Supplementary Table 1 and assay accuracy and precision is shown in Supplementary Table 2.

Pharmacokinetic analysis examining maximal serum concentration as ng/mL(C_max_), time of maximal absorption in hours (T_max_), time half-life elimination in hours (T_0.5 elim_), serum concentration area under the curve as ng*hr/mL (AUC_0- > t_) and mean residence time in hours (MRT), using a pharmacokinetic software package (PK Solutions 2.0, Montrose, CO) for all measurable cannabinoids with sufficient data points for evaluation.

### 2.5 Gastrointestinal transit time

During each trial, each horse was administered 200 0.125 cm-diameter barium-filled low-density polyethylene plastic resin balls (Precision Plastic Ball Company, IL, USA) by nasogastric tube at time 0, as previously described by Sano et al. (29). Every 6 h for 48 h total, all manure in the stall was collected, sealed in a plastic bag, and weighed. At the conclusion of each trial, all plastic bags were radiographed, and the number of barium-filled spheres present in each manure collection was counted (Table 1).

**Table 1:** Median (95% CI) pharmacokinetic parameters after enteral dosing of CBD/CBDA-rich hemp oil in horses using a non-compartmental model.

Analyte	C_max_	T_max_	T_0.5Elim_	AUC	MRT
CBD (2 mg/kg)	5.2 (2.93–8.95)	3.0 (1.52–5.48)	NA	44 (26.52–66.34)	NA
CBD (8 mg/kg)	40.35 (27.70–52.17)	8.0 (4.16–10.34)	8.3 * (6.37–8.93)	501.5 (343.25–687.50)	12.6 (10.29–14.16)
THC (8 mg/kg)	6.65 (5.52–8.25)	6.0 (3.54–9.96)	NA	98.0 (70.74–121.51)	NA
THCA (2 mg/kg)	7.65 (5.32–14.47)	1.25 (0.97–2.63)	10.6 ** (8.58–18.21)	127.0 (100.73–185.3)	12.45 (11.42–13.78)
7-COOH-CBD (2 mg/kg)	133.75 (89.71–169.89)	12.0 (7.87–19.14)	NA	4,450.5 (3206.9–6520.6)	71.4 (57.34–100.44)
7-COOH-CBD (8 mg/kg)	777.5 (630.1–857.4)	12.0 (7.87–19.14)	NA	27,874 (22,851-32,108)	97.1 (73.73–136.82)

Maximum plasma concentration (ng/mL), C_max_; time at maximum plasma concentration (hours), T_max_; elimination half-life (hours), T_0.5Elim_; area under the curve (h*ng/mL), AUC; mean residence time (hours), MRT. *T _0.5 elim_ is based on 6/8 horses with three points beyond Cmax; ** T _0.5 elim_ is based on 7/8 horses with three points beyond Cmax. NA – not applicable due to being a metabolite or horse number for proper analysis being less than 6 horses in the cohort.

### 2.6 Pedometry

To quantify independent movement by the horses, pedometers were placed on the right front and hind pastern. The pedometers (Omron Healthcare, Inc., Hoffman Estates, IL) were used for activity monitoring of foals in a study reported by Grubb et al. (30). The pedometers were secured on the dorsal aspect of the pastern by creating a pocket made of gauze and elastic tape (Elastikon, Johnson & Johnson, NJ, USA), so that each pedometer would stay in place over the course of the study. Measurements were collected starting at time point zero after the baseline neurological evaluation. Subsequent readings were documented before and after gait evaluation at 0.5, 1, 2, 4, 12, and 24 h after treatment so that steps due to forced activity during the gait exam could be subtracted from the total step count.

### 2.7 Vital parameters, hematology and blood chemistry

Horses were weighed on a calibrated scale prior to each trial. Heart rate and respiratory rate were recorded at time 0, 0.5, 1, 2, 4, 12, and 24 h. Blood samples were collected by jugular venipuncture into 10 mL EDTA and heparinized tubes prior to each trial and submitted for hematology and plasma chemistry analysis, respectively, to ensure general health. Heparinized blood samples were collected again 24 h after nasogastric intubation with control or either CBD/CBDA oil treatment for repeat plasma chemistry analysis.

### 2.8 Mentation and gait scoring

Mentation and gait exams were video recorded at times 0, 0.5, 1, 2, 4, 12, and 24 h and reviewed in randomized order by a board-certified large animal internal medicine specialist blinded to the treatment, trial number, and time point. Each exam consisted of observing the horse from a distance undisturbed in the stall, during approach of the handler and interaction with the horse, walking the horse in a straight line viewed from the front, back and either side, turning the horse in tight circles in either direction, and backing the horse. Horses were assigned mentation scores as follows: 0 (bright and alert, normal, appropriate responsiveness to stimuli and environment); 1 (lethargy, somewhat blank facial expression with slight drooping of the ears and eyelids, sluggish responsiveness to stimuli, and reduced voluntary activity); 2 (stupor, stands in one place with the head held low, responds only to strong stimuli); 3 (semi-coma, stuporous and recumbent); and 4 (coma, recumbent and does not respond to any stimulus). The reviewer also provided a “Yes” or “No” response to indicate hyperesthesia, excitability, or ataxia in each video.

### 2.9 Statistics

Data were statistically evaluated using Statistix 10.0 (Analytical Software, Tallahassee, FL). Continuous variables (heart rate, respiratory rate, pedometry data, manure production in kilograms, barium ball recovery (%), pharmacokinetic parameters, and plasma chemistry parameters) were assessed for normality using a Shapiro–Wilk test. Physiologic variables (heart rate and respiratory rate) were compared between treatment groups using a repeated measures ANOVA. Pedometry, plasma chemistry, and mentation and gait score data were compared using a Kruskal-Wallis test. Manure production and cumulative barium ball recovery by time and treatment were evaluated by factorial ANOVA.

## 3 Results

### 3.1 Pharmacokinetics

**Figure 1:**
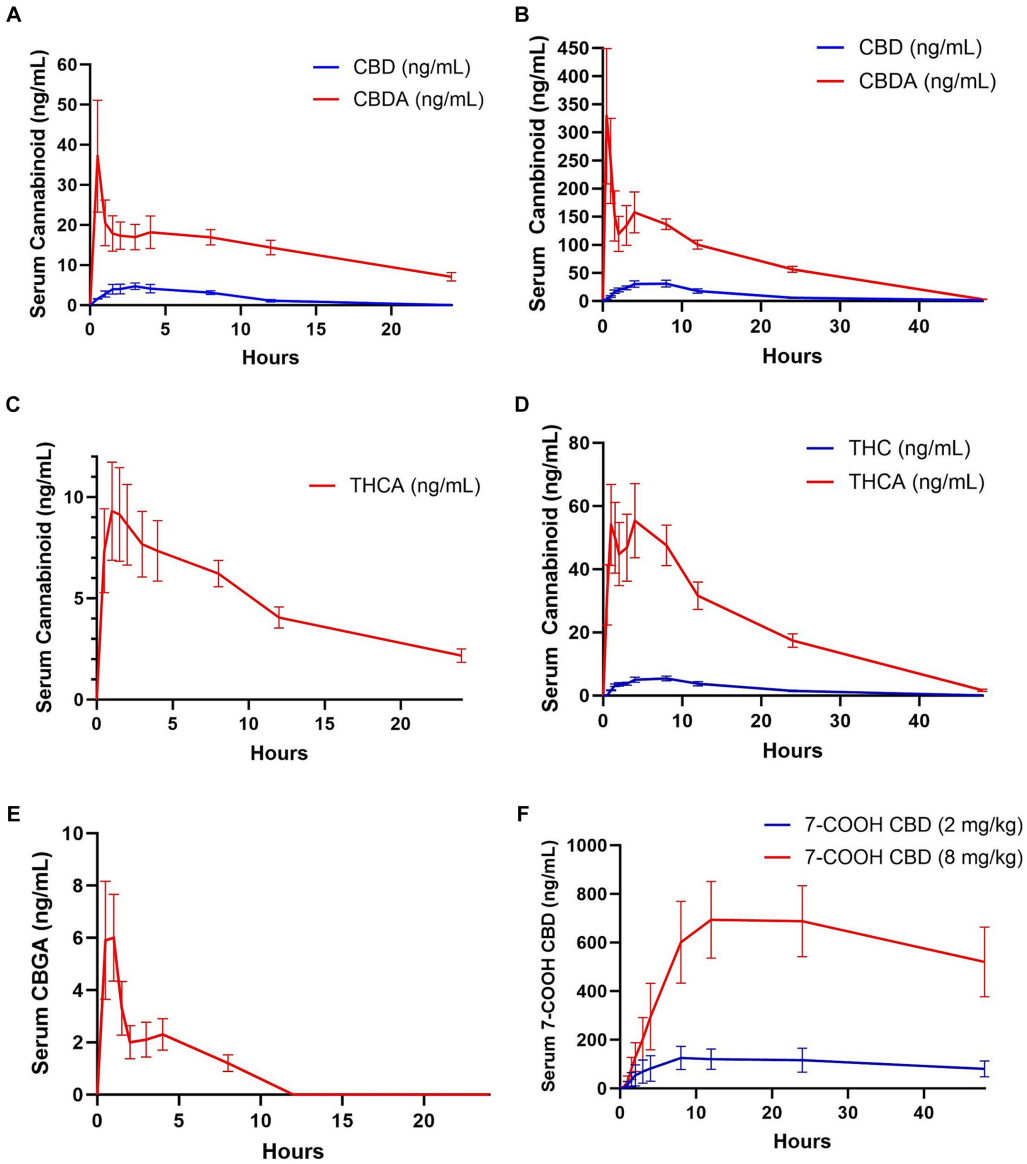
Mean ± standard error analyte concentrations in horses after enteral administration of a single dose of a full spectrum CBD/CBDA-rich hemp oil at 2 or 8 mg/kg doses in a randomized cross-over design. **(A)** CBD and CBDA at 2 mg/kg dose (*n* = 8), **(B)** CBD (*n* = 8) and CBDA (*n* = 8) at 8 mg/kg dose **(C)** THCA at 2 mg/kg dose (*n* = 8) **(D)** THC (*n* = 8) and THCA (*n* = 8) at 8 mg/kg dose **(E)** CBGA at 8 mg/kg dose (*n* = 8), and **(F)** 7-COOH-CBD at 2 and 8 mg/kg dose (*n* = 8).

No cannabinoids were detected in any of the baseline blood samples. Pharmacokinetic parameters could not be determined for CBC, CBCA, CBG, 11-OH, CBN, or 7-OH-CBD due to falling below the quantitation limits for all samples in both groups. THC and CBGA were measured in only a few samples in the 2 mg/kg CBD/CBDA group; all others were below the limit of quantification. CBD serum concentrations were near the lower limit of quantitation (1–2 ng/mL) in 5 of the horses and below the limit of quantitation in 2 horses by 12 h when treated with 2 mg/kg CBD/CBDA treatment. The final horse only had 3 time points with CBD concentration above the lower limit of quantitation thereby only allowing for reporting of Cmax, Tmax and AUC. When treated with 8 mg/kg CBD/CBDA the CBD concentration in the serum was at the lower limit of quantitation (1–2 ng/mL) at 48 h with 4 horses being in that range and 4 horses being below. Due to a later than expected T max (8 h) T_0.5 elim_ and MRT were based on 6 of 8 horses with sufficient data points for analysis and are thus reported. The median maximal concentration of CBD was 40.35 ng/mL and the median half-life of elimination of CBD was 7.75 h in the 8 mg/kg group. The mean concentration-time curve for CBD in all horses at 2 and 8 mg/kg is represented in Figures 1A,B at the respective concentrations.

THC was only quantifiable in four horses in the 2 mg/kg dose group, with quantifiable concentrations at 1–5 timepoints between 1 and 8 h after dosing with concentrations being near the lower limit of quantification at 1–2 ng/mL. THC was measured in all horses in the 8 mg/kg dose group, with a median maximum concentration of 6.65 ng/mL occurring at 6 h. Time to maximum concentration was highly variable, from 2 h in one horse to 12 h in 2 horses. All but one horse had quantifiable THC in the serum at 1.5 h, and all horses were at or near the lower limit of quantification (1.0–2.6 ng/mL) with only one horse being below the limit of quantification at 24 h, and no horses had measurable THC by 48 h. The mean concentration-time curves for THC are represented in Figure 1D for the 8 mg/kg treatment and are not represented for the 2 mg/kg time point as 4 of 8 horses had no measurable THC at any time point with only 4 horses showing between 1 and 2 ng.

CBDA was measurable for pharmacokinetic assessment in both the 2 and 8 mg/kg group however due to a biphasic curve a Tmax1/Tmax 2 and Cmax1 and Cmax 2 are reported as well as AUC and MRT (Table 2). THCA was measurable for pharmacokinetic assessment in 7 of 8 horses in the 2 mg/kg group following typical pharmacokinetic modeling allow for calculations of all parameters including MRT and T_0.5 elim_, while when horses were treated with 8 mg/kg they displayed atypical biphasic Cmax1 and Cmax2 pharmacokinetics in 5 of the 8 horses and are thus reported in Table 2, similar to CBDA results. The mean concentration-time curves for CBDA, THCA, and CBGA for all horses are represented in Figures 1A–E, respectively.

**Table 2:** Median (95% CI) pharmacokinetic parameters of CBDA and THCA after enteral dosing of CBD/CBDA-rich hemp oil in horses using a non-compartmental model.

Analyte	C_maxP1_	T_maxP1_	C_maxP2_	T_maxP2_	AUC	MRT
CBDA (2 mg/kg)	20.05 (4.14–69.76)	0.5 (0.24–1.13)	17.7 (10.12–28.97)	8.0 (4.53–10.05)	410.5 (289.53–560.97)	12.8 (11.49–14.14)
CBDA (8 mg/kg)	312.2 (92.67–614.45)	0.5 (0.43–1.07)	164.45 (115.88–251.54)	4.0 (3.54–7.21)	3,353 (2,753.3–4,006.7)	13.1 (11.03–15.77)
THCA (8 mg/kg)*	76.3 (16.03–121.75)	1.3 (0.97–4.43)	59.3 (38–43-89.97)	4.0 (3.83–7.57)	1,130 (789.2–1,336.72)	14.4 (13.05–15.55)

Maximum plasma concentration of peak 1 (ng/mL), C_maxP1_; time of maximum plasma concentration of peak 1 (hours), T_maxP1_; maximum plasma concentration of peak 2 (ng/mL), C_maxP2_; time of maximum plasma concentration of peak 2 (hours), T_maxP2_; area under the curve (h*ng/mL), AUC; mean residence time (hours), MRT. *THCA C_maxP2_ and T_maxP2_ are data from 5 horses as 3 did not show a “double peak.”

The major metabolite 7-COOH-CBD Cmax was at 12 h for both the 2 mg/kg and 8 mg/kg group at 133.7 mg/mL and 777.5 ng/mL, respectively. The AUC for 7-COOH CBD at 8 mg/kg was 27,874 ng/mL which was 50 fold greater than CBD suggesting very rapid metabolism of CBD (Table 2 and Figure 1F).

### 3.2 Gastrointestinal transit time

**Figure 2:**
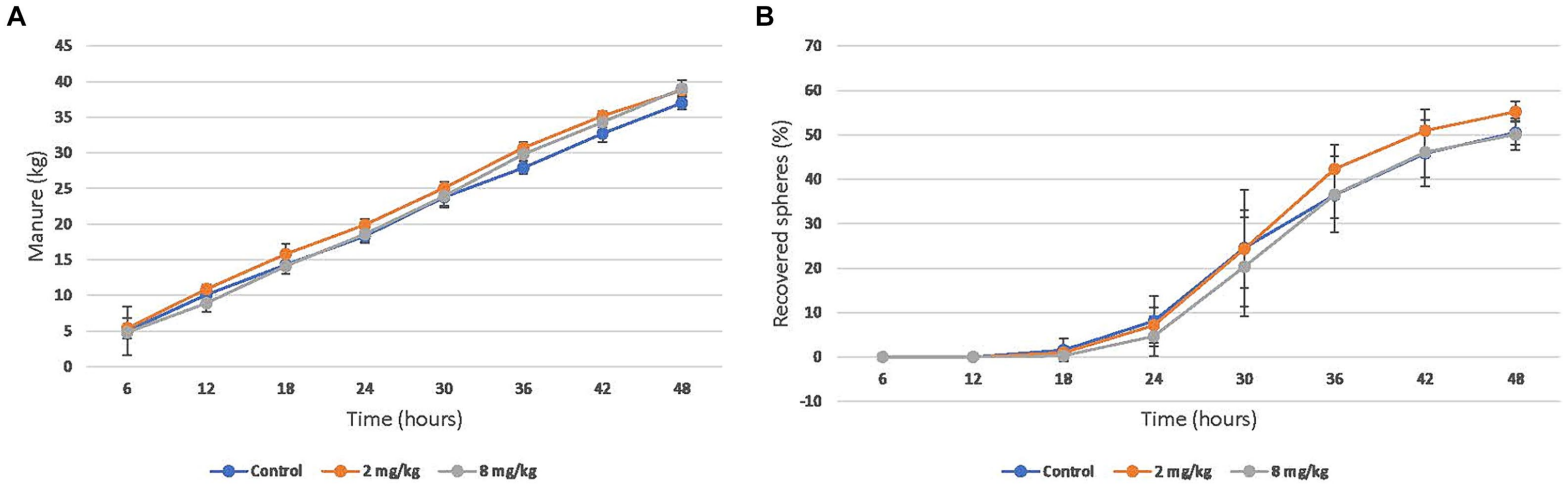
Mean (95% CI) cumulative **(A)** Manure production and **(B)** barium sphere recovery after enteral administration of a single dose of CBD/CBDA rich hemp oil to stall-confined horses (*n* = 8) using placebo (water), 2 mg/kg or 8 mg/kg in a randomized cross-over design.

The mean cumulative manure production over 48 h was 36.9 (SD 9.9) kg in the control group, 38.7 (SD 4.8) kg in the 2 mg/kg group, and 39.1 (SD 9.4) kg in the 8 mg/kg group (Figure 2A). The mean recovery of barium spheres over 48 h was 50.5% (SD 14.6) in the control group, 55.2% (SD 10.3) in the 2 mg/kg group, and 50.1% (SD 14.4) in the 8 mg/kg group (Figure 2B). Neither manure production nor gastrointestinal transit time were significantly different between any of the groups.

### 3.3 Pedometry

**Figure 3:**
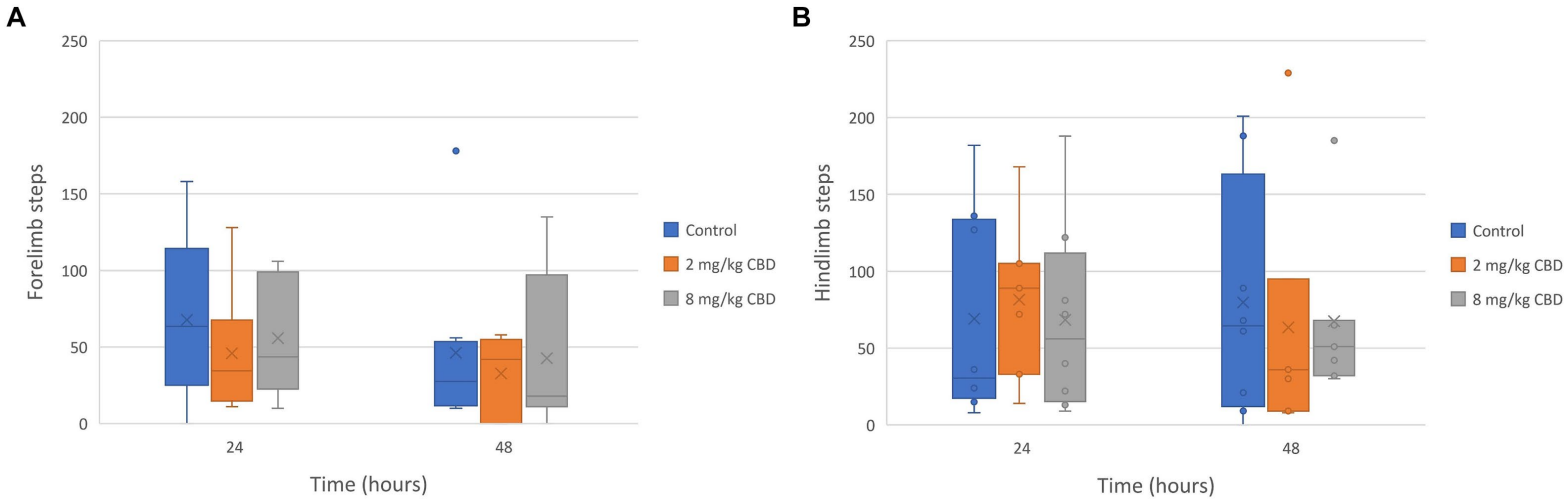
**(A)** Forelimb and **(B)** hindlimb steps taken after enteral administration of a single dose of CBD/CBDA-rich hemp oil to stall-confined horses (*n* = 8) at 2 mg/kg or 8 mg/kg in a randomized cross-over design.

The median, quartile, and range of the number of steps recorded by each horse’s pedometer during the study periods are represented in Figure 3. The median number of forelimb and hindlimb steps recorded by each horse’s pedometers was not significantly different between treatment groups.

### 3.4 Vital parameters, hematology and blood chemistry

All horses were determined to be healthy with no clinically relevant abnormalities on pre-trial hematology and plasma chemistry. There were no significant differences in heart rate or respiratory rate between groups at any time before or following treatments. There were no significant differences between pre-nasogastric intubation time 0 samples and 24-h post samples for any analyte on plasma chemistry in the control group. Median blood urea nitrogen was significantly decreased at 24 h post-dosing (17.5 mg/dL, 95% CI 16.0–18.7) in the 2 mg/kg CBD group compared to the pre-dosing time 0 sample (20.5 g/dL, 95% CI 18.0–21.8; *p* = 0.016). Median glucose was significantly increased at 24 h post-dosing (108 mg/dL, 95% CI 97.8–121.9) in the 8 mg/kg CBD group compared to the pre-dosing time 0 sample (90 mg/dL, 95% CI 85.9–97.4; *p* = 0.003). Median bicarbonate was significantly decreased at 24 h post-dosing (31 mEq/L, 95% CI 29.9–31.1) in the 8 mg/kg CBD group compared to pre-dosing time 0 sample (32 mEq/L, 95% CI 31.2–32.3; *p* = 0.0018). There were no significant differences for any plasma chemistry variable between treatments at each time-point (Supplementary Tables 3, 4).

### 3.5 Mentation and gait scoring

Mentation scores did not differ between the control and treatment groups at any time, and no horse was assigned a mentation score greater than 1 across groups. None of the horses in any treatment group demonstrated hyperesthesia, excitability, or ataxia at any time during the study based on blinded video assessment.

## 4 Discussion

Pharmacokinetic variables for CBD found in this study were comparable to those previously reported in horses, with a relatively short T_max_ and a highly variable C_max_ and elimination half-life (21–26). Though the present study administered CBD oil by nasogastric tube, these C_max_ values are similar to previously reported C_max_ in horses administered oral doses; Ryan et al. reported a C_max_ of 6.14 ng/mL with a 2 mg/kg dose (21), and Yocom et al. reported a C_max_ of 4.3 ng/mL with a 1 mg/kg oral dose and 19.9 ng/mL with a 3 mg/kg oral dose (22). However, the C_max_ reported in the present study is dramatically lower than that reported by Williams et al. who administered 2 mg/kg CBD in a pelleted formulation at the time of feeding and reported a C_max_ of 51 ± 15 ng/mL, after 7 days of dosing which would equate to a dose of 4 mg/kg of our CBD/CBDA rich hemp oil, providing 2 mg/kg of CBD suggesting some level of tissue accumulation being possible with chronic administration (23). Eichler and colleagues showed long term dosing of 3 mg/kg twice daily resulting in Cmax concentrations of only 12 ng/mL after 12 h, while after 2 weeks serum nadir and peak concentrations were between 8 and 50 ng/mL range (26). Twelve senior horses treated with a single oil based 2 mg/kg dose of CBD displayed an average Cmax of approximately 19 ng/mL which is similar to prior results, yet slightly higher, suggesting that age may play a role in metabolism of CBD (24). Sanchez and colleagues studied naked oil preparations of CBD and micellar forms of CBD at 10 mg/kg to assess absorption over 12 h suggesting that AUC for both formulation were similar, yet Cmax was higher in the micellar form of CBD again suggesting that form of CBD deliver may also influence absorption kinetics (25). These data indicate a difference in the bioavailability of the supplements used and/or significantly enhanced absorption when CBD is administered concurrently with feed or as part of the pelleted ration and that bioaccumulation is tissue may occur with CBD in horses. In humans, the intake and composition of a meal has been shown to significantly affect CBD absorption (31, 32). Overall, enteral absorption of CBD in this oil-based formulation was noticeably lower in horses than has previously been reported in dogs. While a 2 mg/kg dose in dogs produced a median peak plasma concentration of 102 ng/mL and nearly 600 ng/mL at the 8 mg/kg dose (9), the median peak plasma concentration in horses was 5.2 ng/mL at the 2 mg/kg dose and only 40.3 ng/mL at the 8 mg/kg dose, therefore more sensitive assays may be required for individual cannabinoids that are detected at the lower limit of quantification for precise results, particularly in the lower dosing.

The present study also evaluated additional cannabinoids, and peak plasma concentrations of CBDA were much higher than those of CBD. Approximately half of the CBD administered in this study was in the form of CBDA (approximately 1 mg/kg in our CBD/CBDA rich hemp dose of 2 mg/kg). These data suggest CBDA may be more bioavailable than CBD in horses. CBDA itself has been associated with antinociceptive and antihyperalgesic effects in rodent models (33, 34), although these effects have yet to be demonstrated in horses. There is very little clinical data on CBDA and its nocioceptive effects, a recent study evaluating CBDA-rich hemp in cows suggested that providing CBDA rich hemp may mitigate stress and make cows more comfortable (11). Also notable are the much higher concentrations of 7-COOH-CBD found in horses in this study in comparison to previous findings in dogs. Dogs administered a 2 mg/kg dose of CBD/CBDA-rich hemp oil in a similar oil-based formulation achieved a Cmax of 13 ± 2 ng/mL and AUC of 159 ± 6 ng/mL, compared to a mean Cmax of 129.8 ± 40 ng/mL and AUC of 4,863 ± 1656.9 ng/mL in horses in the present study. This difference suggests that horses have a higher metabolic capacity for CBD than dogs and cats (35). Our data is very consistent with other studies showing that the major metabolite of CBD in horses is 7-COOH-CBD when dosed at 1–2 mg/kg showing T max in the 8–12 h time range with AUC ranging from 4,000 to 11,500 h*ng/mL with Cmax being between 307 at 2 mg/kg dosing and 85 ng/mL at 1 mg/kg dosing (21, 24).

CBDA, THCA, and CBGA demonstrated biphasic absorption, with two concentration peaks that were more pronounced at the high dose. This “double peak phenomenon” seen with enteric administration of some drugs has been attributed to separate sites of absorption in the gastrointestinal tract, with the absorption limit of the first site determining the magnitude of the second absorption peak (36–38). This phenomenon could also be attributed to enterohepatic recirculation, delayed gastric emptying, or a feeding time phenomenon (39). Double-peak phenomenon is relatively common in horses, and has been demonstrated for phenylbutazone, trimethoprim-sulphachlorpyridazine, and other orally-administered drugs (39, 40).

The doses of CBD evaluated in this study were well-tolerated, with no observable alterations in mentation, activity in stalls or gastrointestinal transit effects. Few statistically significant changes in blood chemistry parameters were noted 24 h after a single administration of 2 mg/kg or 8 mg/kg CBD, and the values fell within normal reference ranges and were not large enough differences to be clinically relevant. However, further research is needed to elucidate the effects of longer-term administration of cannabinoids in this species. While this study only evaluated single doses, Gamble et al. reported increases in alkaline phosphatase in dogs in the fourth week of daily CBD administration (9). Similarly, increases in alanine transaminase consistent with drug-induced liver injury have been reported in healthy human adults after 2–4 weeks of CBD exposure (41). Horses administered CBD demonstrated increases in gamma-glutamyl transferase, aspartate transaminase, and sorbitol dehydrogenase after 6 weeks of administration in a previous study, returning to normal 10 days after discontinuation (22). However, the potential for chronic cannabinoid exposure to cause liver dysfunction or other side effects in horses is currently unknown.

Feed and water intake was free choice in all treatment groups and was not measured. However, no significant differences were observed in either cumulative manure production or in gastrointestinal transit time, and may have affected our pharmacokinetics. Further research may investigate whether the timing and availability of feed and water have an effect on the enteric bioavailability of cannabinoids in horses.

The study and adoption of cannabinoid products in veterinary medicine continues to be face legal hurdles, even though hemp production and distribution of products containing less than 0.3% THC are federally legal in the United States. State regulations may vary considerably between jurisdictions, but a majority of states allow hemp production, distribution and sales. The oil used in the present study contained approximately 0.13% Δ^9^-THC and 0.13% THCA. In final analysis, the major metabolite associated with psychotropic activity, 11-OH-THC, was undetectable. Serum concentrations of THC reached a mean C_max_ of nearly 7 ng/mL with no adverse events, suggesting that the THC levels are safe with this acute dosing. THCA concentrations using similar dosing were nearly 10-fold higher, further supporting that acidic forms of cannabinoids are absorbed better than their decarboxylated forms. Fortunately, THCA is non-psychotropic and thought to be neuroprotective (42).

Given the variability between products, data on the pharmacokinetics of specific formulations is critical information for veterinarians as CBD, THC, and their metabolites are regulated by many racing jurisdictions and competition horse associations. While the product tested falls well within legal levels of THC, this compound is a Class 1 substance under the Association of Racing Commissioners International (ARCI) and a banned substance under the Federation Equestre Internationale (FEI), meaning that no detectable levels are acceptable. All horses in the 8 mg/kg CBD/CBDA group and 2 horses in the 2 mg/kg CBD group had detectable levels of THC in serum. The ARCI also classifies CBD as a Class 2 substance, for which no detectable levels are acceptable, while the FEI classifies CBD as a controlled and specified substance. It is of note, that while THC, CBD and CBDA are rapidly cleared, the half-life of elimination of 7-COOH CBD is long at 52 h for 2 mg/kg CBD and 71.9 h for 8 mg/kg CBD following a single dose. Additionally, it is important to point out that, to date, there has been no formal studies showing efficacy for any indication in horses in placebo blinded studies with the only study on equine behavior being negative, and one case report suggesting alleviation of cribbing behavior (43, 44). Further studies are needed utilizing proper dosing intervals as it is becoming evident that horses may require increased dosing compared to other species for pharmacodynamic effects and that considering the superior absorption of CBDA that further research on CBDA is needed.

## 5 Conclusion

CBD concentrations following enteral administration of CBD oil were low and elimination relatively rapid, while CBDA appeared to be the predominant cannabinoid present with potential therapeutic benefit in horses provided a CBD/CBDA-rich hemp oil. No adverse effects were encountered; however, this was a single dose study. The results of this study can be used to guide bodyweight dosing and dosing interval in future multi-dose studies. Further evaluation of therapeutic effects as well as potential adverse effects in multi-dose studies in horses are warranted.

## Data availability statement

The raw data supporting the conclusions of this article will be made available by the authors, without undue reservation.

## Ethics statement

The animal study was approved by University of Florida Insitutional Animal Care and Use Committee. The study was conducted in accordance with the local legislation and institutional requirements.

## Author contributions

AT: Conceptualization, Data curation, Investigation, Methodology, Writing – original draft, Writing – review & editing. TM: Conceptualization, Data curation, Funding acquisition, Formal analysis, Investigation, Methodology, Supervision, Writing – original draft, Writing – review & editing. AZ: Investigation, Methodology, Supervision, Writing – original draft, Writing – review & editing. BG: Investigation, Methodology, Supervision, Writing – original draft, Writing – review & editing. AL: Data curation, Formal analysis, Investigation, Methodology, Supervision, Writing – original draft, Writing – review & editing. WS: Formal analysis, Methodology, Software, Validation, Writing – original draft, Writing – review & editing. MM: Methodology, Writing – review & editing. DP: Conceptualization, Writing – review & editing. AB: Data curation, Project administration, Writing – original draft, Writing – review & editing. JW: Conceptualization, Methodology, Writing – review & editing.

## References

[1] Elphick MR. The evolution and comparative neurobiology of endocannabinoid signalling. Philos Trans R Soc Lond Ser B Biol Sci. (2012) 367:3201–15. doi: 10.1098/rstb.2011.0394, PMID: 23108540 PMC3481536

[2] Howlett AC. The cannabinoid receptors. Prostaglandins Other Lipid Mediat. (2002) 68-69:619–31. doi: 10.1016/S0090-6980(02)00060-6, PMID: 12432948

[3] Basavarajappa BS. Critical enzymes involved in endocannabinoid metabolism. Protein Pept Lett. (2007) 14:237–46. doi: 10.2174/092986607780090829, PMID: 17346227 PMC1939815

[4] di Marzo V Piscitelli F. The endocannabinoid system and its modulation by phytocannabinoids. Neurotherapeutics. (2015) 12:692–8. doi: 10.1007/s13311-015-0374-6, PMID: 26271952 PMC4604172

[5] Costa B Giagnoni G Franke C Trovato AE Colleoni M. Vanilloid TRPV1 receptor mediates the antihyperalgesic effect of the nonpsychoactive cannabinoid, cannabidiol, in a rat model of acute inflammation. Br J Pharmacol. (2004) 143:247–50. doi: 10.1038/sj.bjp.0705920, PMID: 15313881 PMC1575333

[6] Galiègue S Mary S Marchand J Dussossoy D Carrière D Carayon P. Expression of central and peripheral cannabinoid receptors in human immune tissues and leukocyte subpopulations. Eur J Biochem. (1995) 232:54–61. doi: 10.1111/j.1432-1033.1995.tb20780.x, PMID: 7556170

[7] Brioschi FA di Cesare F Gioeni D Rabbogliatti V Ferrari F D’Urso ES. Oral transmucosal cannabidiol oil formulation as part of a multimodal analgesic regimen: effects on pain relief and quality of life improvement in dogs affected by spontaneous osteoarthritis. Animals. (2020) 10:1505. doi: 10.3390/ani10091505, PMID: 32858828 PMC7552307

[8] Coelho JC Duarte N Bento da Silva A Bronze MR Mestrinho LA. Placebo-controlled trial of daily oral cannabidiol as adjunctive treatment for cats with chronic gingivostomatitis. Animals. (2023) 13:2716. doi: 10.3390/ani13172716, PMID: 37684980 PMC10487179

[9] Gamble L-J Boesch JM Frye CW Schwark WS Mann S Wolfe L. Pharmacokinetics, safety, and clinical efficacy of cannabidiol treatment in osteoarthritic dogs. Front Vet Sci. (2018) 5:165. doi: 10.3389/fvets.2018.00165, PMID: 30083539 PMC6065210

[10] McGrath S Bartner LR Rao S Packer RA Gustafson DL. Randomized blinded controlled clinical trial to assess the effect of oral cannabidiol administration in addition to conventional antiepileptic treatment on seizure frequency in dogs with intractable idiopathic epilepsy. J Am Vet Med Assoc. (2019) 254:1301–8. doi: 10.2460/javma.254.11.1301, PMID: 31067185

[11] Kleinhenz MD Weeder M Montgomery S Martin M Curtis A Magnin G. Short term feeding of industrial hemp with a high cannabidiolic acid (CBDA) content increases lying behavior and reduces biomarkers of stress and inflammation in Holstein steers. Sci Rep. (2022) 12:3683. doi: 10.1038/s41598-022-07795-z, PMID: 35256692 PMC8901777

[12] Campos AC Moreira FA Gomes FV del Bel EA Guimarães FS. Multiple mechanisms involved in the large-spectrum therapeutic potential of cannabidiol in psychiatric disorders. Philos Trans R Soc Lond Ser B Biol Sci. (2012) 367:3364–78. doi: 10.1098/rstb.2011.0389, PMID: 23108553 PMC3481531

[13] Pertwee RG. The diverse CB1 and CB2 receptor pharmacology of three plant cannabinoids: Δ9-tetrahydrocannabinol, cannabidiol and Δ9-tetrahydrocannabivarin. Br J Pharmacol. (2008) 153:199–215. doi: 10.1038/sj.bjp.0707442, PMID: 17828291 PMC2219532

[14] Bisogno T Hanuš L de Petrocellis L Tchilibon S Ponde DE Brandi I. Molecular targets for cannabidiol and its synthetic analogues: effect on vanilloid VR1 receptors and on the cellular uptake and enzymatic hydrolysis of anandamide. Br J Pharmacol. (2001) 134:845–52. doi: 10.1038/sj.bjp.0704327, PMID: 11606325 PMC1573017

[15] Gobira PH Vilela LR Gonçalves BDC Santos RPM de Oliveira AC Vieira LB. Cannabidiol, a *Cannabis sativa* constituent, inhibits cocaine-induced seizures in mice: possible role of the mTOR pathway and reduction in glutamate release. Neurotoxicology. (2015) 50:116–21. doi: 10.1016/j.neuro.2015.08.007, PMID: 26283212

[16] Suryavanshi SV Kovalchuk I Kovalchuk O. Cannabinoids as key regulators of inflammasome signaling: a current perspective. Front Immunol. (2021) 11:613613. doi: 10.3389/fimmu.2020.613613, PMID: 33584697 PMC7876066

[17] Court MH Mealey KL Burke NS Jimenez TP Zhu Z Wakshlag JJ. Cannabidiol and cannabidiolic acid: preliminary in vitro evaluation of metabolism and drug-drug interactions involving canine cytochrome P-450, UDP-glucuronosyltransferase, and P-glycoprotein. Vet Pharmacol Ther. (2024) 47:1–13. doi: 10.1111/jvp.13403, PMID: 37469115

[18] Takeda S Misawa K Yamamoto I Watanabe K. Cannabidiolic acid as a selective Cyclooxygenase-2 inhibitory component in Cannabis. Drug Metab Dispos. (2008) 36:1917–21. doi: 10.1124/dmd.108.020909, PMID: 18556441

[19] Philpot LM Ebbert JO Hurt RT. A survey of the attitudes, beliefs and knowledge about medical cannabis among primary care providers. BMC Fam Pract. (2019) 20:17. doi: 10.1186/s12875-019-0906-y, PMID: 30669979 PMC6341534

[20] Kondrad E Reid A. Colorado family physicians’ attitudes toward medical marijuana. J Am Board Family Med. (2013) 26:52–60. doi: 10.3122/jabfm.2013.01.120089, PMID: 23288281

[21] Ryan D McKemie DS Kass PH Puschner B Knych HK. Pharmacokinetics and effects on arachidonic acid metabolism of low doses of cannabidiol following oral administration to horses. Drug Test Anal. (2021) 13:1305–17. doi: 10.1002/dta.3028, PMID: 33723919

[22] Yocom AF O’Fallon ES Gustafson DL Contino EK. Pharmacokinetics, safety, and synovial fluid concentrations of single-and multiple-dose oral administration of 1 and 3 mg/kg cannabidiol in horses. J Equine Vet. (2022) 113:103933. doi: 10.1016/j.jevs.2022.103933, PMID: 35307550

[23] Williams MR Holbrook TC Maxwell L Croft CH Ientile MM Cliburn K. Pharmacokinetic evaluation of a cannabidiol supplement in horses. J Equine Vet. (2022) 110:103842. doi: 10.1016/j.jevs.2021.103842, PMID: 34923070

[24] Turner SE Knych HK Adams AA. Pharmacokinetics of cannabidiol in a randomized crossover trial in senior horses. Am J Vet Res. (2022) 83:ajvr.22.02.0028. doi: 10.2460/ajvr.22.02.0028, PMID: 35895770

[25] Sánchez de Medina A Serrano-Rodríguez JM Díez de Castro E García-Valverde MT Saitua A Becero M. Pharmacokinetics and oral bioavailability of cannabidiol in horses after intravenous and oral administration with oil and micellar formulations. Equine Vet J. (2023) 55:1094–103. doi: 10.1111/evj.13923, PMID: 36624043

[26] Eichler F Poźniak B Machnik M Schenk I Wingender A Baudisch N. Pharmacokinetic modelling of orally administered cannabidiol and implications for medication control in horses. Front Vet Sci. (2023) 10:1234551. doi: 10.3389/fvets.2023.1234551, PMID: 37621871 PMC10445762

[27] Deabold KA Schwark WS Wolf L Wakshlag JJ. Single-dose pharmacokinetics and preliminary safety assessment with use of CBD-rich hemp nutraceutical in healthy dogs and cats. Animals. (2019) 9:832. doi: 10.3390/ani9100832, PMID: 31635105 PMC6826847

[28] Anderson LL Etchart MG Bahceci D Golembiewski TA Arnold JC. Cannabis constituents interact at the drug efflux pump BCRP to markedly increase plasma cannabidiolic acid concentrations. Sci Rep. (2021) 11:14948. doi: 10.1038/s41598-021-94212-6, PMID: 34294753 PMC8298633

[29] Sano H Martin-Flores M Santos LCP Cheetham J Araos JD Gleed RD. Effects of epidural morphine on gastrointestinal transit in unmedicated horses. Vet Anaesth Analg. (2011) 38:121–6. doi: 10.1111/j.1467-2995.2010.00588.x, PMID: 21303443

[30] Grubb TL Kurkowski D Sellon DC Seino KK Coffey T Davis JL. Pharmacokinetics and physiologic/behavioral effects of buprenorphine administered sublingually and intravenously to neonatal foals. J Vet Pharmacol Ther. (2019) 42:26–36. doi: 10.1111/jvp.12715, PMID: 30242851

[31] Crockett J Critchley D Tayo B Berwaerts J Morrison G. A phase 1, randomized, pharmacokinetic trial of the effect of different meal compositions, whole milk, and alcohol on cannabidiol exposure and safety in healthy subjects. Epilepsia. (2020) 61:267–77. doi: 10.1111/epi.16419, PMID: 32012251 PMC7065230

[32] Silmore LH Willmer AR Capparelli EV Rosania GR. Food effects on the formulation, dosing, and administration of cannabidiol (CBD) in humans: a systematic review of clinical studies. Pharmacotherapy: the journal of human pharmacology and drug. Therapy. (2021) 41:405–20. doi: 10.1002/phar.2512 PMC8485703 33583102

[33] Rock EM Limebeer CL Parker LA. Effect of cannabidiolic acid and ∆9-tetrahydrocannabinol on carrageenan-induced hyperalgesia and edema in a rodent model of inflammatory pain. Psychopharmacology. (2018) 235:3259–71. doi: 10.1007/s00213-018-5034-1, PMID: 30225659

[34] Vigli D Cosentino L Pellas M de Filippis B. Chronic treatment with Cannabidiolic acid (CBDA) reduces thermal pain sensitivity in male mice and rescues the hyperalgesia in a mouse model of Rett syndrome. Neuroscience. (2021) 453:113–23. doi: 10.1016/j.neuroscience.2020.09.041, PMID: 33010341

[35] Wang T Zakharov A Gomez B Lyubimov A Trottier NL Schwark WS. Serum cannabinoid 24 h and 1 week steady state pharmacokinetic assessment in cats using a CBD/CBDA rich hemp paste. Front Vet Sci. (2022) 9:895368. doi: 10.3389/fvets.2022.895368 35937287 PMC9355628

[36] Godfrey KR Arundel PA Dong Z Bryant R. Modelling the double peak phenomenon in pharmacokinetics. IFAC Proc. (2009) 42:127–32. doi: 10.3182/20090812-3-DK-2006.0001 20381191

[37] Weitschies W Bernsdorf A Giessmann T Zschiesche M Modess C Hartmann V. The talinolol double-peak phenomenon is likely caused by presystemic processing after uptake from gut lumen. Pharm Res. (2005) 22:728–35. doi: 10.1007/s11095-005-2588-5 15906167

[38] Mirfazaelian A Mahmoudian M. A simple pharmacokinetics subroutine for modeling double peak phenomenon. Biopharm Drug Dispos. (2006) 27:119–24. doi: 10.1002/bdd.492, PMID: 16400712

[39] Baggot J. Bioavailability and bioequivalence of veterinary drug dosage forms, with particular reference to horses: an overview. J Vet Pharmacol Ther. (1992) 15:160–73. doi: 10.1111/j.1365-2885.1992.tb01003.x, PMID: 1433478

[40] Duijkeren E Vulto AG van Oldruitenborgh-Oosterbaan MMS Kessels BG van Miert AS Breukink HJ. Pharmacokinetics of trimethoprim/sulphachlorpyridazine in horses after oral, nasogastric and intravenous administration. J Vet Pharmacol Ther. (1995) 18:47–53. doi: 10.1111/j.1365-2885.1995.tb00550.x, PMID: 7752306

[41] Watkins PB Church RJ Li J Knappertz V. Cannabidiol and abnormal liver chemistries in healthy adults: results of a phase I clinical trial. Clin Pharmacol Ther. (2021) 109:1224–31. doi: 10.1002/cpt.2071, PMID: 33022751 PMC8246741

[42] Nadal X del Río C Casano S Palomares B Ferreiro-Vera C Navarrete C. Tetrahydrocannabinolic acid is a potent PPARγ agonist with neuroprotective activity. Br J Pharmacol. (2017) 174:4263–76. doi: 10.1111/bph.14019, PMID: 28853159 PMC5731255

[43] Eichler F Ehrle A Machnik M Jensen KC Wagner S Baudisch N. Behavioral observations, heart rate and cortisol monitoring in horses following multiple oral administrations of a cannabidiol containing paste (part 2/2). Front Vet Sci. (2024) 10:1305873. doi: 10.3389/fvets.2023.1305873, PMID: 38234983 PMC10791836

[44] Cunha RZ Felisardo LL Salamanca G Marchioni GG Neto OI Chiocchetti R. The use of cannabidiol as a novel treatment for oral stereotypic behaviour (crib-biting) in a horse. Vet Anim Sci. (2023) 19:100289. doi: 10.1016/j.vas.2023.100289, PMID: 36824298 PMC9941357

