# Supplementary material for: Pharmacokinetics and tolerability of single-dose enteral cannabidiol and cannabidiolic acid rich hemp in horses (Equus caballus)
Source: Front Vet Sci. 2024 Apr 12;11:1356463. doi: 10.3389/fvets.2024.1356463 (PMC11047043; doi:10.3389/fvets.2024.1356463)
Supplement: Supplementary file 1 [file Data_Sheet_1.PDF]

Table 1. LC-MS/MS parameters and calibration curve range in horse serum.

Reference Standard			Internal Standard		Calibration Curve Range (ng mL^{-1})
Name	Retention Time (min)	MRM (Polarity)	Name	MRM (Polarity)	
CBD	4.7	315>193 (+)	CBD-d3	318>196 (+)	1 – 1,000
CBDA	4.35	359>219 (+)	CBD-d3	318>196 (+)	1 - 1,000
THC	5.8	315>193 (+)	THC-d3	318>196 (+)	1 - 1,000
THCA	6.3	357>313 (-)	THCA-d3	360>316 (-)	0.25 - 1,000
CBG	4.6	317>193 (+)	CBD-d3	318>196 (+)	0.5 - 1,000
CBGA	4.5	361>219 (+)	CBD-d3	318>196 (+)	1 - 1,000
CBC	6.1	315>193 (+)	THC-d3	318>196 (+)	2.5 - 1,000
CBN	5.4	311>223 (+)	CBD-d3	318>196 (+)	1 - 1,000
7-COOH-CBD	2.35	345>299 (+)	7-COOH-CBD-d3	348>302 (+)	1 - 5,000
7-OH-CBD	2.5	331>201 (+)	7-OH-CBD-d5	336>201 (+)	10 - 1,000
COOH-THC	3.7	345>299 (+)	COOH-THC-d9	354>308 (+)	1 - 500
COOH-THC-Glu	2.15	521>299 (+)	COOH-THC-Glu-d3	524>302 (+)	1 - 500
11-OH-THC	3.6	331>201 (+)	11-OH-THC-d3	334>201 (+)	2.5 - 1000
