# Supplementary material for: Pharmacokinetics and tolerability of single-dose enteral cannabidiol and cannabidiolic acid rich hemp in horses (Equus caballus)
Source: Front Vet Sci. 2024 Apr 12;11:1356463. doi: 10.3389/fvets.2024.1356463 (PMC11047043; doi:10.3389/fvets.2024.1356463)
Supplement: Supplementary file 2 [file Data_Sheet_2.PDF]

Supplementary Table 2. Mean Accuracy and Precision of Assay.
Analysis was done in duplicate on two days (N = 4).

Nominal Conc. (ng/mL)	CBD		CBDA		THC		CBGA		CBN	
	Accuracy (%)	%CV	Accuracy (%)	%CV	Accuracy (%)	%CV	Accuracy (%)	%CV	Accuracy (%)	%CV
**1**	95.4	8.9%	100.9	4.0%	109.1	11.8%	96.1	12.8%	94.5	20.8%
**2.5**	102.7	8.9%	97.7	10.4%	97.6	5.3%	101.2	11.4%	90.8	6.8%
**5**	100.4	9.4%	103.4	3.6%	91.7	4.4%	97.7	7.4%	101.6	8.9%
**10**	102.9	5.7%	98.7	7.1%	98.7	3.9%	102.8	3.8%	101.9	2.4%
**25**	100.4	2.4%	94.4	4.8%	102.3	5.4%	100.0	4.1%	97.6	9.1%
**50**	101.6	7.1%	102.6	1.4%	100.4	3.6%	102.3	3.4%	102.0	1.4%
**100**	101.9	2.4%	99.8	4.4%	99.3	4.0%	102.5	5.2%	102.4	1.5%
**250**	104.5	5.6%	103.6	4.6%	101.9	3.5%	105.4	6.6%	104.4	1.8%
**500**	102.2	4.3%	99.2	4.4%	103.3	0.8%	101.9	3.6%	99.2	2.7%
**1000**	101.6	4.0%	102.6	3.1%	99.1	2.7%	100.7	3.3%	99.0	0.9%

Nominal Conc. (ng/mL)	THCA		CBG		Nominal Conc. (ng/mL)	7-COOH-CBD		Nominal Conc. (ng/mL)	7-OH-CBD	
	Accuracy (%)	%CV	Accuracy (%)	%CV		Accuracy (%)	%CV		Accuracy (%)	%CV
**0.25**	103.7	2.8%			**1**	100.5	17.4%	**10**	96.1	18.5%
**0.5**	99.0	2.2%	101.0	8.0%	**2.5**	103.0	14.3%	**25**	107.6	12.6%
**1**	96.5	3.7%	98.1	10.4%	**5**	101.0	6.6%	**50**	96.0	10.1%
**2.5**	98.5	2.7%	96.8	7.1%	**10**	100.8	11.0%	**100**	101.6	9.9%
**5**	98.6	2.1%	99.4	6.0%	**25**	94.6	5.0%	**250**	97.7	6.8%
**10**	99.1	3.1%	97.9	0.9%	**50**	92.7	7.8%	**500**	101.2	4.7%
**25**	101.7	3.0%	100.9	1.6%	**100**	95.2	4.4%	**1000**	96.9	5.7%
**50**	104.7	3.1%	103.3	4.1%	**250**	100.2	6.2%			
**100**	103.5	1.0%	102.6	4.5%	**500**	95.8	7.4%			
**250**	105.7	3.8%	106.2	4.1%	**1000**	100.0	2.4%			
**500**	104.8	4.0%	102.6	3.7%	**2500**	105.8	5.0%			
**1000**	99.1	4.1%	98.8	0.8%						

Nominal Conc. (ng/mL)	CBC		11-OH-THC		Nominal Conc. (ng/mL)	COOH-THC		COOH-THC-Glu	
	Accuracy (%)	%CV	Accuracy (%)	%CV		Accuracy (%)	%CV	Accuracy (%)	%CV
**2.5**	104.5	7.5%	97.4	10.4%	**1**	91.6	17.7%	109.2	17.7%
**5**	94.4	13.1%	97.6	17.1%	**2.5**	100.2	16.1%	101.0	11.4%
**10**	96.5	8.4%	97.2	10.2%	**5**	94.2	7.1%	96.4	5.1%
**25**	105.5	7.3%	104.4	11.5%	**10**	97.6	10.5%	99.8	7.5%
**50**	104.9	5.3%	100.3	9.4%	**25**	99.6	3.1%	93.6	11.3%
**100**	99.2	6.8%	99.4	8.9%	**50**	102.9	3.5%	94.4	13.7%
**250**	100.9	9.0%	100.8	5.6%	**100**	106.1	1.3%	101.9	4.4%
**500**	101.9	4.1%	104.6	7.5%	**250**	102.7	2.7%	99.0	7.7%
**1000**	100.9	3.8%	99.9	1.2%	**500**	100.1	3.2%	95.9	5.6%
