# Supplementary material for: Pharmacokinetics and tolerability of single-dose enteral cannabidiol and cannabidiolic acid rich hemp in horses (Equus caballus)
Source: Front Vet Sci. 2024 Apr 12;11:1356463. doi: 10.3389/fvets.2024.1356463 (PMC11047043; doi:10.3389/fvets.2024.1356463)
Supplement: Supplementary file 3 [file Data_Sheet_3.pdf]

Supplementary Table 3. Median (range) pretreatment and 24-hour post-treatment plasma chemistry results from horses (n=8) treated with no treatment (control), 2 mg/kg cannabidiol (CBD) oil, and 8 mg/kg CBD oil by nasogastric tube.

Analyte	Control			2 mg/kg CBD			8 mg/kg CBD		
	Pre-tx	24 hr	*p*	Pre-tx	24 hr	*p*	Pre-tx	24 hr	*p*
CK	311 (244-546)	289 (213-633)	N.S.	287 (220-565)	308 (223-706)	N.S.	362 (234-409)	280 (211-555)	N.S.
AST	250 (196-389)	271 (199-359)	N.S.	283 (224-406)	273 (230-384)	N.S.	278 (213-364)	261 (206-369)	N.S.
GLDH	3 (2-23)	3 (1-21)	N.S.	4 (2-25)	3.5 (1-62)	N.S.	4 (2-12)	2.5 (1-18)	N.S.
GGT	10.5 (8-23)	10 (9-23)	N.S.	11.5 (2-22)	11.5 (10-15)	N.S.	10 (8-14)	10 (8-12)	N.S.
ALP	141 (91-212)	134 (81-202)	N.S.	141 (85-187)	129 (92-188)	N.S.	125 (79-173)	119 (79-171)	N.S.
Total Bilirubin	1.8 (1-4.9)	1.7 (1.2-4.1)	N.S.	1.6 (0.9-3.9)	1.6 (1.1-4.6)	N.S.	1.6 (0.9-4.3)	1.6 (0.9-5.7)	N.S.
Direct Bilirubin	0.5 (0.3-0.8)	0.5 (0.4-0.8)	N.S.	0.4 (0.3-0.7)	0.5 (0.4-0.7)	N.S.	0.5 (0.3-0.7)	0.5 (0.4-0.8)	N.S.
Glucose	89 (81-112)	97 (84-126)	N.S.	92 (83-137)	100 (83-114)	N.S.	**90 (81-101)**	**108 (93-130)**	**0.003**
Triglycerides	25 (13-35)	25 (18-60)	N.S.	26 (17-46)	26 (22-32)	N.S.	26 (16-44)	25 (14-36)	N.S.
Albumin	3.4 (3.2-3.7)	3.5 (3.2-3.6)	N.S.	3.4 12 ((3.2-3.7)	3.4 (3.3-3.6)	N.S.	3.5 (3.2-3.5)	3.4 (3.2-3.7)	N.S.
Globulin	3.1 (2.4-4.1)	3 (2.5-3.8)	N.S.	3.0 (2.7-3.8)	2.9 (2.5-3.9)	N.S.	3.0 (2.5-3.5)	2.8 (2.6-3.3)	N.S.
A:G	1.1 (0.9-1.4)	1.1 (0.9-1.4)	N.S.	1.2 (0.8-1.2)	1.2 (0.8-1.3)	N.S.	1.2 (0.9-1.4)	1.3 (1-1.3)	N.S.
Total Protein	6.5 (5.8-7.8)	6.4 (0.9-7.3)	N.S.	6.5 (6-7.2)	6.3 (5.9-7.2)	N.S.	6.4 (5.9-6.7)	6.4 (5.8-6.6)	N.S.
BUN	18 (17-23)	17 (16-19)	N.S.	**21 (16-23)**	**17.5 (15-19)**	**0.016**	19 (15-27)	17 (15-20)	N.S.
Creatinine	1.4 (1.0-1.6)	1.3 (1.1-1.7)	N.S.	1.3 (1.0-1.5)	1.3 (1.0-1.6)	N.S.	1.3 (1.1-1.5)	1.4 (1.3-1.5)	N.S.
Na+	139 (135-142)	138 (137-142)	N.S.	139 (138-140)	138 (135-140)	N.S.	140 (138-142)	138 (137-140)	N.S.
K+	3.7 (3.1-6.5)	4 (2.7-6.4)	N.S.	3.7 (3.3-6.2)	3.6 (3.1-3.8)	N.S.	3.7 (2.4-4.8)	3.5 (2.6-5.4)	N.S.
Cl-	102 (98-104)	101 (99-103)	N.S.	101 (99-103)	101 (100-102)	N.S.	101 (99-104)	102 (101-103)	N.S.
HCO_3-	31 (28-35)	29 (28-33)	N.S.	32 (29-35)	31 (29-32)	N.S.	**32 (31-32)**	**31 (29-31)**	**0.0018**
Ca^{2+}	11.8 (11.5-12.2)	11.9 (11.5-12.5)	N.S.	11.7 (11-12.4)	12.0 (11.4-12.9)	N.S.	11.6 (11.1-12.3)	11.9 (11.6-12.6)	N.S.
P	3.6 (2.2-4.9)	3.2 (2.8-3.4)	N.S.	3.6 (2.8-4.7)	2.9 (1.9-3.9)	N.S.	4.2 (2.7-4.6)	2.7 (2.1-3.8)	N.S.
Mg	2.2 (1.7-2.5)	2.1 (1.9-2.8)	N.S.	2.2 (1.5-3)	2.1 (1.9-2.9)	N.S.	2.2 (1.7 – 3.3)	2.1 (1.9-2.7)	N.S.
Anion Gap	11.3 (7.9-11.8)	10.7 (8.6-11.8)	N.S.	10.9 (8.1-12.3)	10.0 (97.4-12.0)	N.S.	10.5 (9.6-12.7)	9.5 (7.8-10.5)	N.S.

* Significant difference between pre-treatment (pre-tx) and 24-hour post-treatment (24 hr) values within a treatment using Kruskall-Wallis ($p<0.05$). N.S. – Not significant.
