# Supplementary material for: Pharmacokinetics and tolerability of single-dose enteral cannabidiol and cannabidiolic acid rich hemp in horses (Equus caballus)
Source: Front Vet Sci. 2024 Apr 12;11:1356463. doi: 10.3389/fvets.2024.1356463 (PMC11047043; doi:10.3389/fvets.2024.1356463)
Supplement: Supplementary file 4 [file Data_Sheet_4.pdf]

Supplementary Table 4. Mean (± SE) heart rate and respiratory rate in horses (n=8) treated with no treatment control, 2 mg/kg CBD, or 8 mg/kg CBD by nasogastric tube.

**Time** (hours)	**Heart Rate** Control	**Heart Rate** 2 mg/kg CBD	**Heart Rate** 8 mg/kg CBD	*p*	**Respiratory Rate** Control	**Respiratory Rate** 2 mg/kg CBD	**Respiratory Rate** 8 mg/kg CBD	*p*
0	40 ± 1.7	37 ± 1.7	39 ± 1.5		15 ± 1.5	17 ± 0.5	15 ± 1.0	
0.5	39 ± 2.0	39 ± 1.1	37 ± 1.0		16 ±1.2	17 ± 1.5	14 ± 1.2	
1	38 ± 0.7	38 ± 2.2	38 ± 1.3		17 ± 1.0	17 ±1.4	13 ± 0.9	
2	38 ± 0.7	36 ± 1.3	39 ± 0.8	0.325	16 ± 0.9	15 ± 2.8	13 ± 0.7	0.496
4	38 ± 0.7	38 ± 1.3	36 ± 0.9		15 ± 1.1	17 ± 1.4	12 ± 0.7	
12	39 ± 1.7	41 ± 2.1	37 ± 1.6		16 ± 0.9	18 ± 3.4	14 ± 1.3	
24	37 ± 1.4	37 ± 1.3	37 ± 0.9		17 ±1.4	11 ± 1.3	16 ± 1.1	

Repeated measures ANOVA, treatment*time, $p<0.05$.
